# Increased expression of CD70 in relapsed acute myeloid leukemia after hypomethylating agents

**DOI:** 10.1007/s00428-024-03741-8

**Published:** 2024-02-22

**Authors:** Mario L. Marques-Piubelli, Bijender Kumar, Rafet Basar, Siler Panowski, Surabhi Srinivasan, Kevin Norwood, Sacha Prashad, Victoria Szenes, Arun Balakumaran, Akanksha Arandhya, Wei Lu, Khaja Khan, Daniela Duenas, Salome McAllen, Javier A Gomez, Jared K. Burks, Sunil Acharyal, Gautam Borthakur, Wei-Lien Wang, Wei Wang, Sa Wang, Luisa M. Solis, David Marin, Katayoun Rezvani, May Daher, Francisco Vega

**Affiliations:** 1 Department of Translational Molecular Pathology, The University of Texas MD Anderson Cancer Center, 1515 Holcombe Blvd, Houston, TX 77030 USA; 2 Department of Stem Cell Transplantation and Cellular Therapy, The University of Texas MD Anderson Cancer Center, 1515 Holcombe Blvd, Houston, TX 77030 USA; 3 Allogene Therapeutics, South San Francisco, CA USA; 4 Department Hematopathology, The University of Texas MD Anderson Cancer Center, 1515 Holcombe Blvd, Houston, TX 77030 USA; 5 Department of Leukemia, The University of Texas MD Anderson Cancer Center, 1515 Holcombe Blvd, Houston, TX 77030 USA; 6 Department of Pathology, The University of Texas MD Anderson Cancer Center, 1515 Holcombe Blvd, Houston, TX 77030 USA

**Keywords:** CD70, relapsed acute myeloid leukemia, HMA, routine diagnostic technique

## Abstract

Acute myeloid leukemia (AML) is the most common acute leukemia in adults. While induction chemotherapy leads to remission in most patients, a significant number will experience relapse. Therefore, there is a need for novel therapies that can improve remission rates in patients with relapsed and refractory AML. CD70 is the natural ligand for CD27 (a member of the TNF superfamily) and appears to be a promising therapeutic target. Consequently, there is considerable interest in developing chimeric antigen receptor (CAR) T-cell therapy products that can specifically target CD70 in various neoplasms, including AML. In this study, we employed routine diagnostic techniques, such as immunohistochemistry and flow cytometry, to investigate the expression of CD70 in bone marrow samples from treatment-naïve and relapsed AML patients after hypomethylating agents (HMA). Also, we evaluated the impact of HMA on CD70 expression and examined CD70 expression in various leukemic cell subsets and normal hematopoietic progenitors.

**Supplementary Information:**

The online version contains supplementary material available at 10.1007/s00428-024-03741-8.

## Introduction

Acute myeloid leukemia (AML) represents the most common acute leukemia in adults, and despite of the recent advances in biological targets, the treatment remains challenging, especially in relapsed settings [1]. Although most patients achieve complete remission after initial therapy, relapses may occur in up to 50% of younger and in the majority of older patients, contributing for an overall dismal prognosis [2]. Hypomethylating agents (HMA), decitabine and azacytidine, with venetoclax are currently the standard of care for patients with AML who are not eligible for intensive chemotherapy [3].

The clinical results of chimeric antigen receptor (CAR) T-cell therapy in AML are disappointing [4]. Major barriers to success include shared expression of the target antigen (e.g., CD33 and CD123) on AML cells and normal hematopoietic stem cells (HSCs) thus increasing the risk of marrow aplasia, and heterogeneous expression or absence of target antigens on blasts, predisposing to leukemia escape [5].

CD70 is a member of the tumor necrosis factor (TNF) superfamily and the ligand for the cytokine receptor CD27 [6]. CD70 expression is usually transient, tightly regulated, and restricted to a small subset of activated T, B, and dendritic cells, and is not essential for a functional immune system [7]. CD70 has been reported to be upregulated in AML and to contribute to myeloid blast stemness [8]. CD27 seems to be increased in serum samples of AML patients with unfavorable prognosis [9–11].

Preclinical results from early phase clinical trials targeting CD70 with monoclonal antibodies or CAR T-cell therapy in AML have shown promising results [1, 9, 12, 13]. Although some studies have previously evaluated the CD70 expression in solid tumors and in lymphomas [14, 15], the evaluation of CD70 expression in AML patients in a clinical setting has not been explored. In this study, we investigate the expression of CD70 using immunohistochemistry (IHC), flow cytometry (FC), and dual immunofluorescence (IF), in bone marrow samples from treatment-naïve and relapsed AML patients after HMA. Also, we evaluated the impact of HMAs on CD70 expression and examined CD70 expression in various leukemic cell subsets and normal hematopoietic progenitors.

## Material and methods

We retrospectively evaluated the expression of CD70 in AML patients who relapsed after first line therapy with azacitidine and/or decitabine and had available formalin-fixed paraffin-embedded (FFPE) samples (bone marrow clot specimens with adequate number of marrow particles) at MD Anderson Cancer Center between January, 2011, and July, 2020. This study was performed under an approved IRB protocol and patient waiver consent. The samples were distributed and initially evaluated in three different cohorts: discovery cohort (DC), flow cohort (FC), and IF cohort (IFC). The DC included patients with available paired FFPE (treatment-naïve and after relapse) bone marrow clots for IHC evaluation. The FC was composed of the same patients with available fresh bone marrow aspirates for flow cytometry testing, and the IFC also included the same patients with remaining FFPE bone marrow clot samples that were analyzed by dual IF for CD34/CD70 and IHC. All the methods and reagents including cell lines and primary blasts used are described in the Supplementary Table 1.

## Results and discussion

Eighty-two samples from 41 patients were included in DC, while 16 samples were available for FC and 8 for IFC. The median age of the patients included in the DC was 72 years (range: 40–91) and most of the patients were male (*n* = 22, 53%) and Caucasian (*n* = 35, 86%) with no previous history of malignancy (*n* = 23, 56%). The median blast percentage was 40% in the treatment-naïve samples and 13% in the relapsed samples. At the time of diagnosis, a diploid karyotype or deletion of 5/5q and/or 7/7q was the most common cytogenetic abnormalities, while *TET2*, *DNMT3A*, and *NPM1* were the most common mutations. The median time to relapse and overall survival was 12 months (range: 3–41) and 24 months (95%CI: 18.1–24.8), respectively, and most of the patients had expired at the last follow-up (*n* = 38, 92%) (Supplementary Tables 2 and 3).

**Fig. 1:**
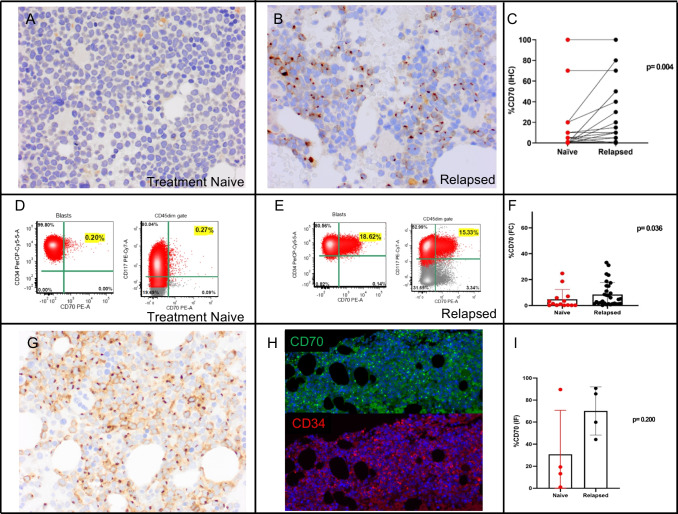
Examples of CD70 expression as detected by immunohistochemistry (IHC) and flow cytometry (FC) in treatment naïve and relapsed AML patients. **A**, **B** In this case, IHC for CD70 in a bone marrow clot was negative at diagnosis (**A**) and detected in about 50% of the blasts at the relapse one year later (**B**); a paired analysis of naïve and relapsed samples shows a significantly higher expression in the relapsed group (*p* = 0.004) (**C**). **D**, **E** FC of a naïve treated sample (**D**) shows expression of CD70 in 0.20% of the CD34+ blasts and 0.27% of CD117+ blasts, while at time of the relapsed demonstrates positivity for CD70 in 18.6% of the CD34+ blast population and in 15.3% of the CD117+ (**E**); an analysis using FC to compare naïve and relapsed samples shows a statistically significant higher expression in relapsed samples (*p* = 0.036) (**F**). **G**, **H** IHC for CD70 in a bone marrow clot of a newly diagnosed AML shows positivity in most of the blasts (**G**) and the dual immunofluorescence (IF) for CD34 (red)/CD70 (green) in the same specimen shows positivity in 89.6% of the blasts as quantified by digital imaging (**H**). A comparison using dual IF showed a higher expression in relapsed samples in comparison with naïve treated, although not statistically significant, likely due to the low number of samples with double IHC and IF staining (**I**). Note that the immunostaining pattern includes staining of the cytoplasmic membrane and a Golgi/paranuclear stain in most of positive blasts. Blasts with only a Golgi stain pattern are also seen, and if these blasts express CD70 at the level of the cellular membrane is unknown currently

Among the DC cases, 34% (13/38) of the naïve-treated and 39% (15/38) of the relapsed group were positive for CD70 at any level (more than 1%). In the naïve treated group six cases (15.8%) have at least 10% blasts positive for CD70 as detected by IHC. In the relapsed group, 12 of 38 (31.6%) have at least 10% blasts positive for CD70. In three cases from each naïve and relapsed group, there was no remaining bone marrow particles. The expression of CD70 in the DC cases was heterogeneous in both naïve-treated and relapsed groups and ranged from 0 to 100% of positivity in the blasts (Supplementary Table 3). The relapsed group showed a significantly higher expression of CD70 when compared to the naïve group (mean in DC: 6.5% vs. 13%, *p* = 0.004; mean in FC: 4.8% vs. 8.4%, *p* = 0.036) (Fig. 1).

When comparing the CD70 expression with different clinicopathologic variables, there was a significantly higher expression of CD70 in female patients (*p* < 0.0001, median: 97% vs. 0%) with no history of malignancy (*p* < 0.0001, median: 100% vs. 0%) in the samples of the relapsed group of the DC. Treatment-naïve samples did not show any correlation with clinicopathologic variables.

Although IHC has limitations due the subjective estimation of CD70 positivity in the blast compartment, there was a satisfactory concordance between IHC and flow cytometry and IHC and dual IF, respectively [*R* squared of 0.4899 (*p* = 0.003) and 0.9436 (*p* < 0.0001), respectively]. Although not statistically significant in cases evaluated by IF from IFC due the sample size, there was also a higher expression of CD70 in the relapsed group (median: 72.7% vs. 16.3%, *p* = 0.200) (Fig. 1I). To further investigate if the percentage of blasts had any correlation with the CD70 expression, we performed a linear regression. The *R* squared in the treatment-naïve and relapsed groups was, respectively, 0.0003 (*p* = 0.905) and 0.08 (*p* = 0.073), which indicates a low level of concordance. Therefore, CD70 expression does not correlate with the blast percentage present in the sample.

**Fig. 2:**
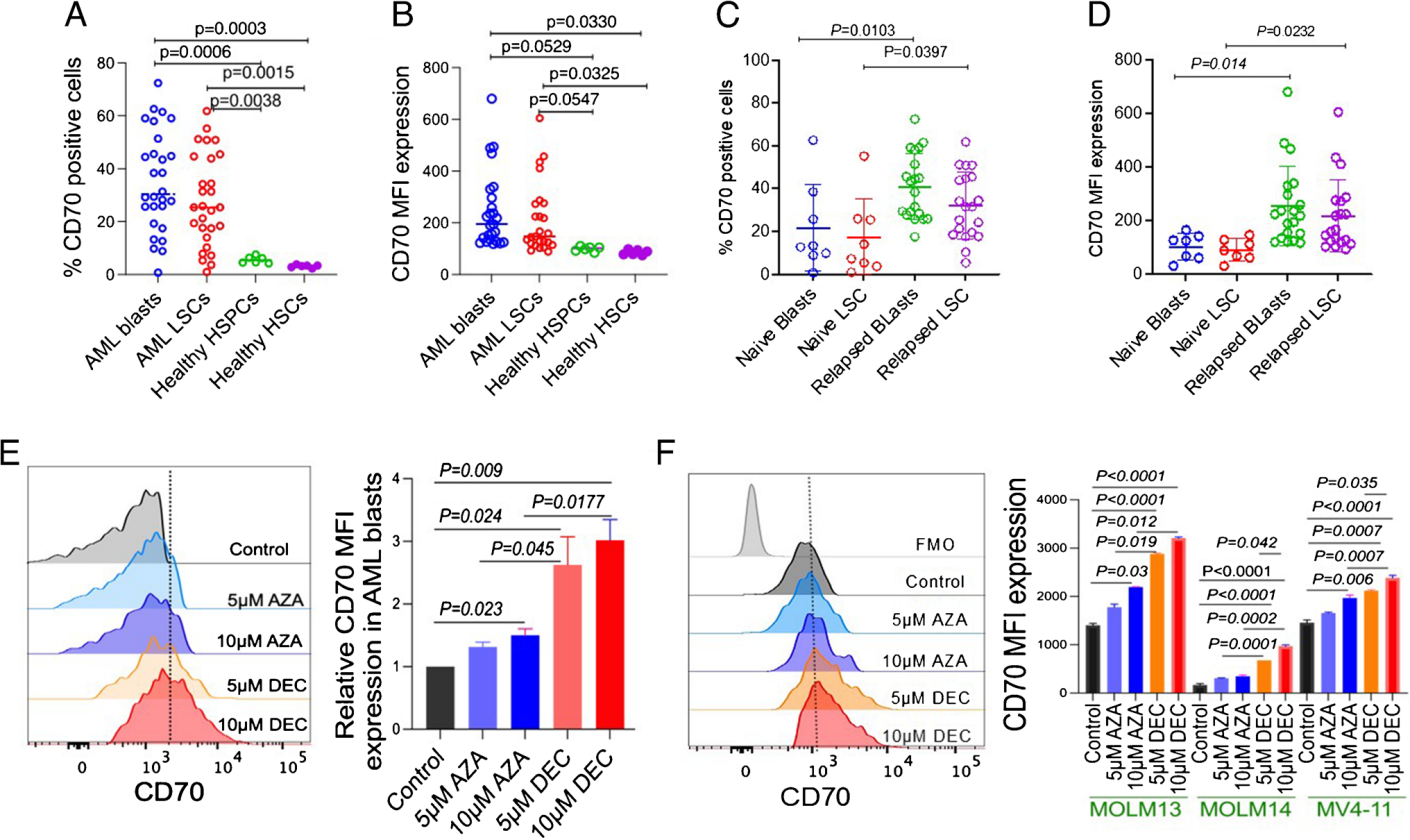
CD70 expression is significantly increased in patient-derived AML blasts and leukemic stem cells (LSC), as well as in relapsed patients following exposure to HMA. **A**, **B** Graphs showing the percentage of CD70 positivity (**A**) and mean fluorescent intensity (MFI) (**B**) on AML blasts and LSCs compared to healthy HSPCs and HSCs. **C**, **D** Graphs showing CD70 percentage (**C**) and CD70 MFI (**D**) on AML blasts and LSCs of treatment naïve and relapsed AML patients. **E** CD70 expression as detected by FC in untreated, 5-azacytidine (AZA)-, and decitabine (DEC)-treated AML blasts for 24 h. Left panel shows representative CD70 expression histograms; right panel shows relative CD70 MFI expression from three naïve AML patients after HMA treatments. **F** CD70 expression and CD70 MFI expression in three AML cell lines (MOLM13, MOLM14, and MV4-11) before and after treatment with AZA and DEC for 24 h. Left panel shows representative CD70 expression histograms; right panel shows combined CD70 expression data from all the AML cell lines

To corroborate the CD70 expression data obtained by flow cytometry and to further dissect CD70 expression in the various leukemic subsets, we evaluated bone marrow clot samples from 28 additional patients (validation cohort). The patient characteristics for this cohort are summarized in Supplementary Table 2. CD70 expression is significantly higher on bulk leukemic blast population and on leukemic stem cells (LSCs) compared to normal bone marrow hematopoietic stem cells (HSCs) and hematopoietic progenitor cells (HSPCs), as evaluated by percentage expression and by mean fluorescent intensity (MFI) (Fig. 2A, B). Consistent with results from the DC, AML blasts and LSCs from relapsed patients had significantly higher CD70 expression compared to those from newly diagnosed patients who are treatment naïve (*p* = 0.014 and p=0.023, respectively, Fig. 2C, D). AML bulk blasts were defined as CD45 dim lin-CD34^−^ CD38^+/−^ CD33^+/high^ CD117^+/−^, and AML LSCs were defined CD45 dim lin-CD34+CD38+/− CD117^+^ CD123^+^ CD33^+/−^; LSCs were further functionally defined by serial re-plating colony formation unit (CFU) assay (Supplementary Figure).

Interestingly, in a recent phase I/II clinical trial study published by Riether and colleagues, targeting CD70 with cusatuzumab (a high-affinity anti-CD70 monoclonal antibody) showed high rates of elimination of LSC in patients treated with azacitidine and that azacitidine induces CD70 expression in LSC by demethylating the CD70 promoter [9]. To further investigate the association between HMA therapy and CD70 expression, we treated three primary AML blasts (naïve) and three AML cell lines (MOLM13, MOLM14, and MVA-11) with 5-azacytidine and decitabine in vitro for 24 h and detected a significant increase in CD70 expression (Fig. 2E, F). Decitabine-treated AML blasts and cell lines exhibited a significant CD70 expression increase compared to 5-azacytidine at similar drug concentrations (Fig. 2E, F).

We acknowledge some limitations in our study, which includes a small number of patients, single-center evaluation, retrospective analysis, and not uniform distribution of clinicopathologic variables.

In conclusion, this study represents an assessment of CD70 expression in clinical AML samples, revealing a prevalence of CD70 in nearly 40% of AML patients at the time of diagnosis although usually a low level of expression. Moreover, our data demonstrate the reliability of detecting CD70 expression through FC (ideally) and by IHC (retrospectively, when needed) with concordant results. Additionally, we found a significant increase in CD70 expression in relapsed AML samples compared to treatment-naïve samples. Notably, we observed a noteworthy association between HMA therapy and the upregulation of CD70 expression in AML blasts. Finally, we identified varying levels of CD70 expression in distinct leukemic cell subsets, as well as in normal hematopoietic stem cells and progenitors. These findings contribute to the growing body of evidence supporting CD70 as a promising target for immunotherapy at least in a subset of cases of relapsed AML following HMA treatment.

## Supplementary information

ESM 1 **Supplementary figure**. A-C) Definitions of bulk blasts and LSCs, normal HSCs and HSPCs: gating strategy for bulk blasts and LSCs for bone marrow samples of AML patients (A), serial replating CFU assay to further characterize LSC population (B), and gating strategy for normal HSC and HSPC (C). (PPTX 1425 kb)

ESM 2 (DOCX 19 kb)

ESM 3 (DOCX 19 kb)

ESM 4 (DOCX 26 kb)
