# Supplementary figures and images for: Increased expression of CD70 in relapsed acute myeloid leukemia after hypomethylating agents
Source: Virchows Arch. 2024 Feb 22;485(5):937–41. doi: 10.1007/s00428-024-03741-8 (PMC11564407; doi:10.1007/s00428-024-03741-8)

## Slide 1
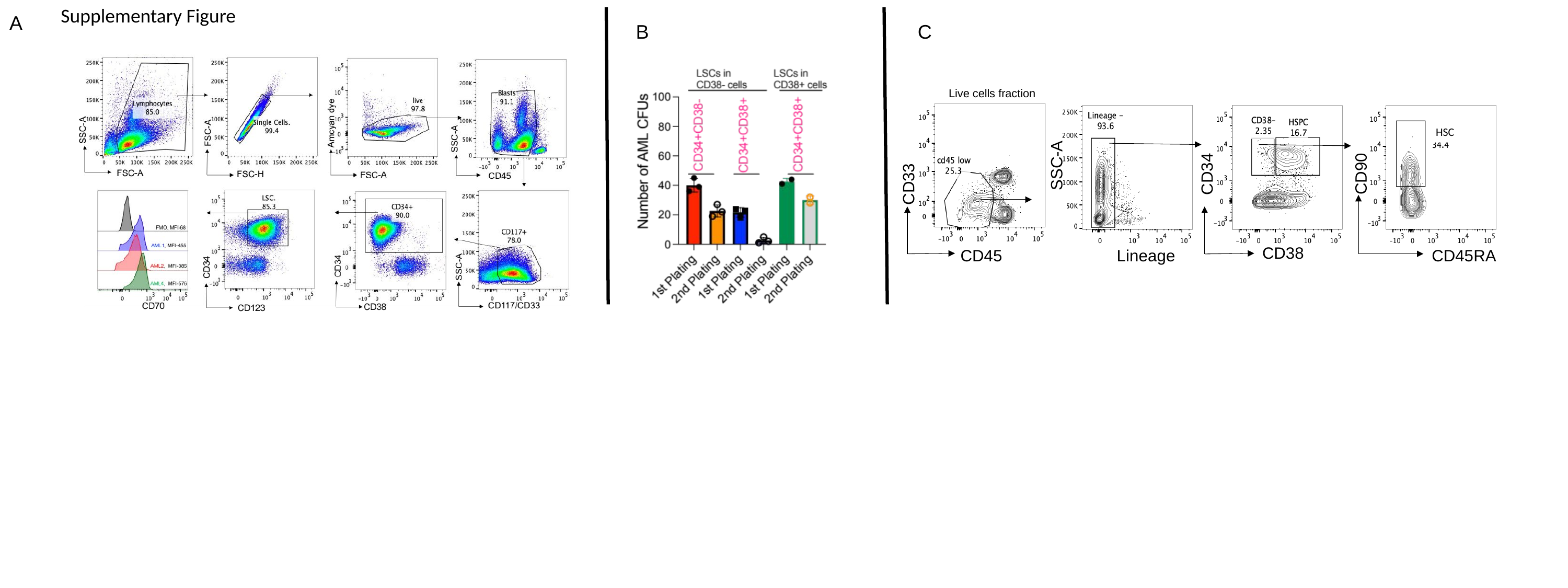

Supplementary Figure
A
D
B
C
Live cells fraction
SSC-A
CD90
CD34
CD33
CD38
CD45
CD45RA
Lineage
HSC

Supplement: Supplementary file 1 — Supplementary figure. A-C) Definitions of bulk blasts and LSCs, normal HSCs and HSPCs: gating strategy for bulk blasts and LSCs for bone marrow samples of AML patients (A), serial replating CFU assay to further characterize LSC population (B), and gating strategy for normal HSC and HSPC (C). (PPTX 1425 kb) [file 428_2024_3741_MOESM1_ESM.pptx]
