# Supplementary material for: Increased expression of CD70 in relapsed acute myeloid leukemia after hypomethylating agents
Source: Virchows Arch. 2024 Feb 22;485(5):937–41. doi: 10.1007/s00428-024-03741-8 (PMC11564407; doi:10.1007/s00428-024-03741-8)
Supplement: Supplementary file 2 — (DOCX 19 kb) [file 428_2024_3741_MOESM2_ESM.docx]

**Supplementary Table 1**

| *Assessment of baseline characteristics* | - Age - Sex - Ethnicity - History of malignancy (solid or hematological tumors) - Percentage of blasts - Karyotype - Molecular profile - Overall survival - Status at last-follow-up |
| --- | --- |
| *Flow Cytometry* | - Fresh bone marrow aspirates   - Minimum: 200k events - Standard staining and lyse/wash technique (PharmLyseTM, BD Biosciences, San Diego, CA, USA) - Panel   - CD34   - CD45   - CD117   - CD70 (Biolegend CAT#355110) - Analysis: FCS Express   - AML LSC: CD45 dim lin-CD34+CD38+/- CD117+ CD123+ CD33+/-   - AML bulk blasts: CD45 dim lin-CD34-CD38+/- CD33 high CD117+/-   - Normal HSC: CD45 dim lin-CD34+CD38- CD90+ CD45RA-   - Normal HSPC: CD45 dim lin-CD34+CD38+ |
| *Dual Immunofluorescence (IF)* | - Deparaffinization and antigen retrieval: 107°C (15 min)   - Biogenex EZ-Retriever (MW015-IR)   - IR SystemEZ-AR 2 Elegans (HX032YCX-GP) buffer - Tissue incubation with primary antibodies at 4C (RTU for CD34 and 1:50 for CD70) - Incubation of fluorescent-labeled secondary antibodies   - Donkey anti-Rabbit IgG (H+L) Highly Cross-Adsorbed Secondary Antibody   - Alexa Fluor™ 488 Cat # A-21206 (green) against CD70 and Goat anti-Mouse IgG (H+L) Cross-Adsorbed Secondary Antibody   - Alexa Fluor™ 647 Cat # A-21235 (red) against CD34 - Whole tissue imaging: Vectra Polaris Multispectral Imaging System (PerkinElmer) version 1.0.10 - Digital image analysis (algorithm stablishing thresholds for positivity in single marker): Visiopharm (Denmark) |
| *Immunohistochemistry (IHC)* | - Leica BOND™ RX autostainer and BOND™ Polymer Refine Detection kit (Leica Biosciences, DS9800) - 4-micron FFPE bone marrow clots - CD70 (clone E3Q1A, cat#69209, 1:50) - Epitope retrieval: ER2 - Analysis: standard microscopy   - Manual scoring under microscope: % positivity in blasts (10% increments)   - Evaluated by two pathologists (MLM-P and FV) |
| *Methods for Methylcellulose CFU Assay* | - Fresh AML bone marrow using ARIAII flow sorter (BD biosciences, Franklin Lakes, NJ)   - CD45low CD34+CD38-   - CD45low CD34+CD38+ - 5x104 flow sorted cells cultured in a 24 wells plate in semisolid methylcellulose-IMDM media supplemented with   - Exogenous recombinant human SCF and IL-6 (50ng/ml each)   - IL-3   - FLT3-L   - TPO and GCSF (10ng/ml concentration for each) cytokines cocktail   - 3 Units/ml Erythropoietin(EPO) at 37 °C in a humidified atmosphere at 5% CO2 - AML colony-forming units (CFU) scored microscopically (day 14) - Retrieved cells replated in fresh methylcellulose media and CFU analyzed again after 2 weeks culture in both CD38 positive and CD38 negative fractions |
| *Statistical analysis* | - Differences in continuous variables   - Mann-Whitney or Kruskal-Walls test - Comparison of different methods   - Linear regression - Overall survival   - Time interval from the start of therapy to death or last follow-up   - Kaplan-Meier - p value ≤.05 (two-tailed) considered statistically significant - SPSS 21 (IBM SPSS Statistics, IBM Corporation) and GraphPad Prism 8 (GraphPad Software) |
| *Antibodies used* | - PE anti-human CD70 Antibody, cat#355104, Biolegend - PE/Dazzle™ 594 anti-human CD123 Antibody, cat#396708, Biolegend - APC Mouse Anti-Human CD34, cat#555824, BD Bioscience - PE-Cy™7 Mouse Anti-Human CD38, cat#560677, BD Bioscience - BV711 Mouse Anti-Human CD45RA, cat#563733, BD Bioscience - BUV395 Mouse Anti-Human CD33, cat#740293, BD Bioscience - PE/Cyanine5 anti-human CD90 Antibody, cat#328112, Biolegend - APC-Cy™7 Mouse Anti-Human CD45, cat#557833, BD Bioscience - Alexa Fluor® 700 anti-human CD117 (c-kit) Antibody, cat#313246, Biolegend - FITC anti-human CD235a (Glycophorin A) Antibody, cat#349104, Biolegend - FITC Mouse Anti-Human CD3, cat#561802, BD Bioscience - FITC Mouse Anti-Human CD19, cat#555412, BD Bioscience - FITC Mouse Anti-Human CD14, cat#557153, BD Bioscience - LIVE/DEAD™ Fixable Violet Dead Cell Stain Kit, for 405 nm excitation, cat# L34955, Thermo Fisher |
| *Genomics of the AML patients used for the in vitro studies* | - AML   - Diploid   - Mutation: *NPM1, IDH2, SRSF2* - AML   - t(11;19)(q23;p13)   - *MLL* rearranged   - Mutation: *K-RAS* - AML   - Complex Karyotype   - Mutation: *RUNX1* |
