# Supplementary material for: Increased expression of CD70 in relapsed acute myeloid leukemia after hypomethylating agents
Source: Virchows Arch. 2024 Feb 22;485(5):937–41. doi: 10.1007/s00428-024-03741-8 (PMC11564407; doi:10.1007/s00428-024-03741-8)
Supplement: Supplementary file 3 — (DOCX 19 kb) [file 428_2024_3741_MOESM3_ESM.docx]

**Supplementary Table 2**. Baseline characteristics of patients diagnosed with Acute Myeloid Leukemia (AML) who relapsed after Azacytidine and/or Decitabine and its association with CD70 expression in the naïve and relapsed bone marrow specimens of discovery cohort and in the validation cohort.

|  | **Discovery Cohort, n= 41 (%)** | **Validation Cohort, n=28 (%)** |
| --- | --- | --- |
| **Age, median (range), years** | 72 (40 – 91) | 58 (20 – 84) |
| **Sex*** |  |  |
| Male | 22 (53) | 17 (61) |
| Female | 19 (47) | 11 (39) |
| **Ethnicity** |  |  |
| Caucasian | 35 (86) | 19 (68) |
| African-American | 2 (5) | 6 (21) |
| Hispanic | 2 (5) | 1 (3.6) |
| Asian | 1 (2) | 1 (3.6) |
| Other | 1 (2) | 1 (3.6) |
| **History of Malignancy*** |  |  |
| No | 23 (56) | 18 (64) |
| Yes | 18 (44) | 10 (36) |
| **% Blasts, mean (range, SD)** |  |  |
| Naïve | 40% (1-89%, 0.26) | 8 (29) |
| Relapsed | 13% (5-91%, 0.27) | 20 (71) |
| **CD34 positivity (FC)** | 41 (100) | 28 (100) |
| **Karyotype at diagnosis** |  |  |
| Diploid | 24 (62) | 3 (11) |
| -5/5q and/or -7/7q | 5 (13) | 8 (29) |
| Miscellaneous | 5 (13) | 7 (25) |
| Trisomy 8 | 2 (6) | 5 (18) |
| inv16 | 1 (3) | 1 (4) |
| -20q | 1 (3) | 0 (0) |
| Complex | 0 (0) | 9 (32) |
| **Molecular testing, number of mutations** |  |  |
| *ABL* | 2 (1.6) | 0 (0) |
| *APC* | 1 (0.8) | 0 (0) |
| *ANKDR26* | 1 (0.8) | 0 (0) |
| *ASLX1* | 6 (4.8) | 1 (4) |
| *ASLX2* | 0 (0) | 4 (14) |
| *BCOR* | 2 (1.6) | 1 (4) |
| *BCORL1* | 0 (0) | 2 (7) |
| *BRINP3* | 0 (0) | 1 (4) |
| *CBL* | 0 (0) | 3 (11) |
| *CBLC* | 4 (3.2) | 0 (0) |
| *CEBPA* | 8 (6.4) | 2 (7) |
| *CREBBP* | 0 (0) | 2 (7) |
| *DDX4* | 1 (0.8) | 0 (0) |
| *DNMT3A* | 8 (6.4) | 4 (14) |
| *ELANE* | 1 (0.8) | 0 (0) |
| *ETV6* | 0 (0) | 1 (4) |
| *EZH2* | 3 (2.4) | 2 (7) |
| *FLT3 ITD* | 0 (0) | 2 (7) |
| *FLT3 TKD* | 0 (0) | 1 (4) |
| *GATA2* | 0 (0) | 3 (11) |
| *GATA3* | 2 (1.6) | 0 (0) |
| *IDH1* | 5 (4) | 1 (4) |
| *IDH2* | 6 (4.8) | 4 (14) |
| *JAK2* | 7 (5.6) | 1 (4) |
| *KDM6A* | 1 (0.8) | 0 (0) |
| *KIT* | 3 (2.4) | 2 (7) |
| *KRAS* | 0 (0) | 3 (11) |
| *MET* | 1 (0.8) | 0 (0) |
| *MPL* | 2 (1.6) | 0 (0) |
| *NF1* | 2 (1.6) | 3 (11) |
| *NOTCH1* | 4 (3.2) | 1 (4) |
| *NPM1* | 8 (6.4) | 1 (4) |
| *NRAS* | 10 (8) | 4 (14) |
| *PHF6* | 0 (0) | 3 (11) |
| *PIGA* | 2 (1.6) | 0 (0) |
| *PTEN* | 1 (0.8) | 0 (0) |
| *PTPN1* | 1 (0.8) | 0 (0) |
| *RAD21* | 0 (0) | 3 (11) |
| *RUNX1* | 4 (3.2) | 4 (14) |
| *SF3B1* | 1 (0.8) | 1 (4) |
| *SH2B3* | 0 (0) | 1 (4) |
| *SETBP1* | 1 (0.8) | 1 (4) |
| *SMC1A* | 0 (0) | 1 (4) |
| *SRSF2* | 0 (0) | 1 (4) |
| *STSG2* | 0 (0) | 1 (4) |
| *SUZ12* | 0 (0) | 1 (4) |
| *TERC* | 0 (0) | 1 (4) |
| *TERT* | 2 (1.6) | 0 (0) |
| *TET2* | 13 (15) | 1 (4) |
| *TP53* | 3 (2.4) | 7 (25) |
| *U2AF1* | 2 (1.6) | 1 (4) |
| *U2AF2* | 0 (0) | 2 (7) |
| *WT1* | 1 (0.8) | 1 (4) |
| **Median time to relapse (range), months** | 12 (3 – 41) | N/A |
| **Median Overall Survival (95%CI), months** | 24 (18.1 – 24.8) | N/A |
| **Status at last follow-up** |  |  |
| Death | 38 (92) | 18 (64) |
| Alive | 3 (8) | 10 (36) |

*Statistically significant correlation in relapsed cases (p<0.0001)

History of malignancy refers to a range of solid tumors, such as prostate cancer, thymic carcinoma, breast ductal and lobular cancer (both in situ and invasive), bladder cancer, and follicular lymphoma, among others. These cancers had been diagnosed at different times, spanning a period of 3 to 40 years prior to the AML diagnosis. Most patients with these malignancies underwent chemotherapy or radiotherapy treatments.
