# Supplementary material for: Increased expression of CD70 in relapsed acute myeloid leukemia after hypomethylating agents
Source: Virchows Arch. 2024 Feb 22;485(5):937–41. doi: 10.1007/s00428-024-03741-8 (PMC11564407; doi:10.1007/s00428-024-03741-8)
Supplement: Supplementary file 4 — (DOCX 26 kb) [file 428_2024_3741_MOESM4_ESM.docx]

**Supplementary Table 3**. Expression of CD70 by immunohistochemistry in naïve and relapsed samples of patients with Acute Myeloid Leukemia (AML) who relapsed after Azacytidine and/or Decitabine.

| Case | %Blasts (naïve)† | %Blasts CD70+ (naïve) † | %Blasts (relapsed) † | %Blasts CD70+ (relapsed) † | Age (yrs) | Sex | Ethnicity | Hix of Malignancy‡ | Karyotype^+^ | Molecular Testing^+^ | TR (mos) | OS (mos) | SLFU |
| --- | --- | --- | --- | --- | --- | --- | --- | --- | --- | --- | --- | --- | --- |
| 1 | 73% | 0% | 17% | 0% | 73 | M | W | Y | Dip | ***ASXL1*  *IDH2*  *CEBPA* (2)  *TET2* | 10 | 27 | DOD |
| 2 | 60% | 100% | 5% | 100% | 67 | F | W | N | Dip | ***CEBPA*  *DNMT3A*  *NPM1*  *NRAS*  *TET2* | 41 | 50 | DOD |
| 3 | 39% | 0% | 12% | 10% | 89 | M | W | Y | inv16 | ***KIT*  *MPL*  *NRAS* | 8 | 12 | DOD |
| 4 | 30% | 0% | 7% | 0% | 71 | M | W | N | Dip | ****DDX4*  *ELANE*  *NF1*  *NRAS*  *PIGA*  *RUNX1* | 24 | 27 | AWD |
| 5 | 48% | 5% | 84% | 10% | 40 | F | W | Y | Tri 8 | ****ANKDR26*  *ASXL1*  *CBLC*  *GATA3*  *IDH1*  *NOTCH1*  *PIGA*  *PTEN*  *SETBP1*  *TET2* | 8 | 11 | DOD |
| 6 | 80% | 1% | 10% | 5% | 72 | M | W | Y | Dip | ****BCOR*  *NRAS*  *RUNX1*  *U2AF1* | 27 | 35 | DOD |
| 7 | 25% | 0% | 7% | 0% | 74 | M | W | N | -5/5q- and -7/7q- | ****CBLC*  *CBPA*  *DNMT3A*  *TP53* (2) | 10 | 18 | DOD |
| 8 | 85% | 0% | 75% | 50% | 69 | F | W | N | Dip | ****BCOR*  *DNMT3A*  *GATA3*  *IDH1*  *KDM6A*  *NRAS*  *TERT*  *TET2*  *U2AF1* | 9 | 24 | DOD |
| 9 | 77% | 0% | 12% | 0% | 76 | M | W | Y | Dip | ****CBLC*  *SF3B1*  *TET2* | 7 | 14 | DOD |
| 10 | 28% | 1% | 10% | 1% | 70 | M | W | Y | -7/7q- | ****EZH2*  *RUNX1*  *TET2* | 18 | 24 | DOD |
| 11 | 2% | 5% | 20% | 0% | 75 | M | W | N | -5/5q- | ****CBLC*  *NF1*  *TERT*  *TP53*  *WT1* | 4 | 8 | DOD |
| 12 | 21% | N/A* | 8% | 0% | 84 | M | W | N | N/A | N/A | 16 | 27 | DOD |
| 13 | 39% | N/A* | 8% | 0% | 72 | M | W | N | Dip | ***NRAS* | 12 | 22 | DOD |
| 14 | 71% | 5% | 11% | 5% | 72 | F | W | N | Dip | ***IDH2*  *NPM1* | 20 | 55 | DOD |
| 15 | 41% | 10% | 14% | 15% | 72 | F | W | N | N/A | N/A | 18 | 55 | DOD |
| 16 | 28% | 0% | 10% | 0% | 61 | M | W | Y | Misc | **Neg | 9 | 11 | DOD |
| 17 | 25% | 0% | 10% | 0% | 63 | M | W | Y | -5/5q- and -7/7q- | **Neg | 9 | 13 | DOD |
| 18 | 40% | N/A* | 6% | 0% | 69 | F | W | Y | -5/5q- and -7/7q- | **Neg | 17 | 22 | DOD |
| 19 | 76% | 0% | 91% | 50% | 81 | F | H | N | Dip | **Neg | 12 | 14 | DOD |
| 20 | 85% | 0% | 38% | 0% | 71 | F | W | N | Dip | ***APC*  *MET* | 7 | 10 | DOD |
| 21 | 32% | 70% | 41% | 70% | 64 | M | W | N | Misc | N/A | 6 | 6 | DOD |
| 22 | 16% | 0% | 13% | 0% | 81 | M | W | N | Misc | ***Neg | 22 | 42 | DOD |
| 23 | 32% | 0% | 11% | 0% | 83 | F | W | Y | Dip | ****KIT* | 17 | 24 | DOD |
| 24 | 20% | 0% | 14% | 0% | 66 | M | W | N | Dip | **Neg | 17 | 34 | DOD |
| 25 | 81% | 0% | 89% | 0% | 74 | M | W | Y | Dip | ***IDH2*  *JAK2* | 8 | 8 | DOD |
| 26 | 75% | 0% | 42% | 30% | 75 | M | W | N | Dip | ***DNMT3A*  *IDH1* | 6 | 7 | DOD |
| 27 | 20% | 20% | 9% | 40% | 70 | M | W | Y | Dip | ***JAK2*  *NRAS* | 14 | 20 | DOD |
| 28 | 28% | 0% | 25% | N/A* | 68 | F | W | Y | Dip | ** *CEBPA*  *MPL*  *TET2* | 21 | 32 | DOD |
| 29 | 40% | 0% | 21% | 0% | 70 | F | W | N | 20q- | ***ABL*  *ASXL1*  *CEBPA*  *JAK2*  *TET2* | 13 | 28 | DOD |
| 30 | 89% | 0% | 15% | 0% | 72 | M | W | N | Misc | ***IDH2*  *JAK2* | 22 | 26 | DOD |
| 31 | 22% | 0% | 87% | 20% | 66 | F | W | Y | Dip | ***NPM1*  *PTPN1* | 36 | 38 | DOD |
| 32 | 1% | 0% | 18% | 0% | 74 | M | W | N | Dip | ***EZH2*  *IDH1*  *JAK2*  *RUNX1* | 7 | 11 | DOD |
| 33 | 40% | 20% | 60% | 80% | 79 | M | As | Y | Dip | **Neg | 36 | 38 | DOD |
| 34 | 21% | 0% | 13% | N/A* | 91 | F | W | Y | N/A | N/A | 20 | 24 | DOD |
| 35 | 42% | 0% | 9% | 0% | 77 | F | AA | N | Dip | ***CEBPA*  *JAK2*  *NOTCH1*  *NRAS*  *TET2* | 8 | 25 | DOD |
| 36 | 85% | 0% | 8% | 0% | 72 | F | W | N | Dip | ***ABL*  *CEBPA*  *KIT*  *NPM1*  *TET2* | 7 | 15 | DOD |
| 37 | 32% | 1% | 11% | N/A* | 60 | F | W | N | Misc | ** *ASXL1*  *IDH2*  *NOTCH1*  *NRAS* | 3 | 16 | DOD |
| 38 | 73% | 0% | 55% | 0% | 61 | F | O | N | Dip | ***IDH1*  *IDH2*  *NPM1*  *TET2* | 14 | 72 | AWD |
| 39 | 36% | 0% | 6% | 0% | 69 | F | W | Y | 8 | ***DNMT3A*  *EZH2*  *NOTCH1*  *NPM1* | 5 | 6 | DOD |
| 40 | 75% | 1% | 62% | 0% | 74 | M | H | Y | Dip | ** *DNMT3A*  *JAK2*  *NPM1*  *NRAS*  *TET2* | 9 | 10 | AWD |
| 41 | 86% | 10% | 66% | 10% | 75 | F | AA | N | Dip | ** *ASXL1* (2)  *DNMT3A* (2)  *NPM1*  *TET2* | 17 | 17 | DOD |

† Percentage of blasts expressing CD70, manually scored under microscope

‡ Included any type of malignancy (solid or hematological tumors)

^+^Test performed at initial diagnosis

*Absence of bone marrow particles in clot specimen.

**Next generation sequencing panel with 28 genes

*** Next generation sequencing panel with 81 genes

Abbreviations: AA: African-american; As: Asian; AWD: alive with disease; Dip: Diploid; DOD: dead of disease; F: Fale; H: Hispanic; M: male; Misc: Miscellaneous; mos: months; N: No; N/A: not available; Neg: negative; O: Other; OS: overall survival; SLFU: status at last follow-up; TR: time to relapse; Tri8: Trisomy 8; W: white/caucasian; Y: Yes; yrs: years
